# Development of hybrid monoliths incorporating metal–organic frameworks for stir bar sorptive extraction coupled with liquid chromatography for determination of estrogen endocrine disruptors in water and human urine samples

**DOI:** 10.1007/s00604-022-05208-6

**Published:** 2022-02-07

**Authors:** S. Zatrochová, H. Martínez-Pérez-Cejuela, M. Catalá-Icardo, E. F. Simó-Alfonso, I. Lhotská, D. Šatínský, J. M. Herrero-Martínez

**Affiliations:** 1 Department of Analytical Chemistry, Faculty of Pharmacy in Hradec Králové, Charles University, Ak. Heyrovského 1203, Hradec Králové, 500 05 Czech Republic; 2 Department of Analytical Chemistry, University of Valencia, Dr Moliner 50, Burjassot, 46100 Valencia, Spain; 3 Instituto de Investigación Para La Gestión Integrada de Zonas Costeras, Campus de Gandía, Universitat Politècnica de València, C/ Paranimf 1, Grao de Gandía, 46730 Valencia, Spain

**Keywords:** Estrogens, Hybrid monolith, Metal–organic framework, PTFE magnet, Stir bar; Extraction, HPLC-fluorescence detection

## Abstract

A novel coating based on hybrid monolith with metal–organic framework (MOF) onto conventional Teflon-coated magnetic stir bars was developed. For this purpose, the external surface of the Teflon stir bar was firstly vinylized in order to immobilize a glycidyl methacrylate (GMA)–based polymer onto the magnet. Then, an amino-modified MOF of type MIL-101 (NH_2_-MIL-101(Al)) was covalently attached to the GMA-based monolith. After the synthesis process, several parameters affecting extraction of target estrogens by stir bar sorptive extraction (SBSE) including pH, ionic strength, extraction time, stirring rate, desorption solvent, and desorption time were also investigated. The resulting hybrid monolith was evaluated as SBSE sorbent for extraction of three estrogens (estrone, 17β-estradiol, estriol) and synthetic 17β-ethinylestradiol from water and human urine samples followed by HPLC with fluorescence detection (excitation and emission wavelengths, 280 and 310 nm, respectively). Under the optimal experimental conditions, the analytical figures of the method were established, achieving satisfactory limits of detection in the range of 0.015–0.58 µg L^−1^, recovery results ranging from 70 to 95% with RSD less than 6%, and precision values (intra- and inter-extraction units) below 6%.

**Graphical abstract:**

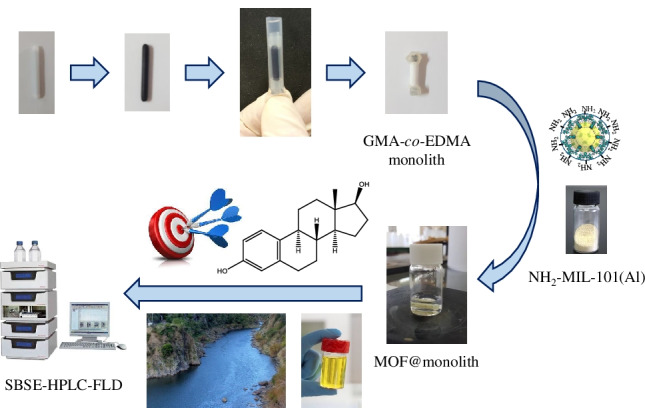

**Supplementary Information:**

The online version contains supplementary material available at 10.1007/s00604-022-05208-6.

## Introduction

Estrogens are known as endocrine-disruptor compounds (EDCs) widely spread in the aquatic system and other environments. Indeed, the attendance- and endocrine-disrupting potency of these molecules in waters, soil, and food are 10,000 times higher than common synthetic chemicals, for example pesticides [1–3]. Estrone (E1), 17β-estradiol (E2), 17β-ethinylestradiol (EE2), and estriol (E3) are the most extended estrogens in the environment and their origin can be various (human urine and feces, cattle activities, aquaculture, among others) [4]. For instance, these target analytes have been detected in environmental and wastewater samples [5–7].

The negative impact of EDCs on human health, aquatic life, and agricultural setting causes problems with infertility, animal hermaphrodism, and other serious health issues. Besides, the EDC levels in biological fluids are strongly related with certain diseases (breast and prostate tumors, infertility, etc.) [8]. Consequently, their presence and evaluation in environmental and biological matrices is of great relevance for safety and health [9, 10].

High-performance liquid chromatography (HPLC) combined with different detectors (such as UV, fluorescence, and MS) is one of the most common technique for determination of these contaminants in biological and environmental samples [2, 11–13]. However, the detection of trace levels of these compounds in complex samples is a challenging task, and a sample pretreatment and efficient preconcentration step is often required. In this sense, several environmental-friendly sample pretreatment techniques have been applied for the determination of estrogens such as liquid-phase microextraction, solid-phase microextraction, magnetic solid-phase extraction (MSPE), and stir bar sorptive extraction (SBSE) [2, 14–18]. In particular, SBSE has many merits such as high sensitivity, good reproducibility, and short extraction time. However, the main drawback of this technique is that most of available commercial coatings are based on polydimethylsiloxane (PDMS) [19, 20], which exhibited limited selectivity, slow extraction kinetics, and low extraction efficiency for these compounds. Therefore, the need for developing novel and advanced stir bar coating materials with a high affinity toward estrogens, thus improving the selectivity and widening the applicability of SBSE, is of great interest [21].

In last years, metal–organic frameworks (MOFs) have received extensive attention due to their fascinating structures and wide range of applications [22, 23]. These crystalline porous materials are made up of coordination bonds between multidentate organic linkers and transition-metal cations that show promising features (e.g., high porosity and surface areas, tailorable functionalities, and large stability). MOFs have been used as catalysts or for gas storage and also in chromatographic area and sample preparation [24, 25]. Despite the good features of these materials, they show the same limitations of powder lab-made materials in sample preparation such as problematical packing into SPE cartridges, tedious centrifugation, or filtration steps needed in dispersive SPE [26]. Besides, in other formats based on fiber/stir bar, supports showed drawbacks such as the fiber fragility [25] and long extraction times (in the range of hours) [27]. In this sense, the development of MOF composites prepared by integration with monoliths is a promising alternative to face up these analytical challenges. This combination allows to incorporate the best features of both materials as the previous mentioned MOF characteristics and porous polymer monoliths advantages (easy in situ preparation, permeability, variable chemical properties, and large chemical stability).

The incorporation of MOF to polymer monoliths can be accomplished by adopting several strategies. The most common way implies the direct embedding of MOF particles into monolithic matrix [28, 29]. Despite the ease of this approach, some limitations such as a low dispersibility of MOFs in the polymerization mixture as well as the sedimentation phenomena under long polymerization times (particularly in thermal initiation) were reported. Alternatively, several approaches have been adopted to attach MOFs on monolithic substrates, including layer-by-layer strategy [30] and covalent bonding of MOFs [31]. This latter alternative presents short preparation time, avoiding tedious sequential cycles, and lower consumption of organic solvents.

In this study, a novel coating based on hybrid monolith with amino modified MIL-101(Al) onto conventional PTFE magnetic stir bars was developed. For this purpose, the PTFE stir bar surface was firstly vinylized in order to immobilize a glycidyl methacrylate (GMA)-based polymer onto the magnet. After a careful selection of MOF, the NH_2_-MIL-101(Al) was covalently attached onto the surface of GMA monolith. The resulting hybrid monolith was evaluated as SBSE coating to extract EDCs (E1, E2, EE2, and E3), including the optimization studies of extraction parameters and evaluation of analytical sorbent features covering breakthrough volume and reusability. The proposed method was successfully applied to the extraction of estrogens in environmental water and urine matrices followed by HPLC-fluorescence detection (FLD).

## Experimental

### Reagents and materials

All the reagents and materials used in this work were of analytical grade unless otherwise stated. More information can be found in the Electronic supplementary material (ESM).

### Instrumentation

All the details regarding characterization of materials and chromatographic conditions of EDCs are reported in ESM.

### Modification of PTFE magnet surface and preparation of monolith-modified magnet

**Fig. 1:**
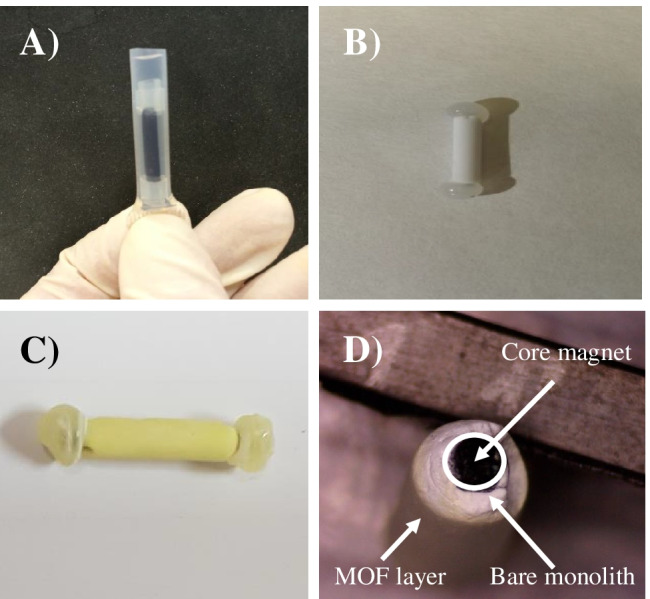
Optical images from experimental assembly (before (**A**) and after monolith polymerization (**B**)), MOF@monolith magnets from lengthwise (**C**), and top view (**D**). Magnification × 20

The chemical modification of magnets was adapted from previous studies [32, 33]. Briefly, it consists of two main steps: (a) the magnet was treated with a commercial etchant solution (Fluoroetch®) to produce hydroxyl groups onto the Teflon surface, and (b) vinylization of the surface with glycidyl methacrylate (GMA) was carried out under the following reaction conditions (GMA 2 M in DMF containing 5 mM triethylamine (pH 8.0), 60 °C for 2 h). The resulting double bonds on the magnet surface behave as linking points during monolith polymerization. The next step is the preparation of a monolith-modified magnet, which is preceded by the preparation of a polymerization mixture. This mixture consists of GMA (32 wt%), EDMA (8 wt%), cyclohexanol (55.7 wt%), 1-dodecanol (4.3 wt%), and LPO (0.3 wt%) (in respect to monomers). The prepared mixture is introduced in a FEP tube (used as vessel) containing the vinylized magnet (see Fig. 1A). After the polymerization reaction (70 °C for 24 h) is done, the magnet is released by cutting the FEP tube, and the magnet coated with polymer monolith (thickness ca. 1 mm) was obtained. The resulting magnet was washed with MeOH and water and air-dried.

### Synthesis of NH_2_-MIL-101 materials

NH_2_-MIL-101(Al) was prepared according to procedure developed elsewhere by Martínez-Pérez-Cejuela et al. [34]. In short, aluminum chloride (482 mg), 2-aminoterephtalic acid (543.45 mg), and DMF (40 mL) were dissolved together in ultrasonic bath. Subsequently, it was placed in a Teflon-lined reactor and the mixture was heated at 130 °C for 72 h. After cooling the reactor to room temperature, the yellow product was filtrated and washed with DMF. Next, the synthesized MOF was washed with ethanol and dried in oven at 60 °C. Other MOF materials (NH_2_-MIL-101(Cr) and NH_2_-MIL-101(Fe)) were also prepared following the procedure previously reported by Martínez-Pérez-Cejuela et al. [34].

### Incorporation of amino-modified MOF to monolithic stir bar

The incorporation of amino-modified MOFs to the magnets with GMA monolith was carried out as follows. Fifty milligrams of NH_2_-MIL-101 (Al) was previously weighed and dispersed in 25 mL DMF. Then, the dispersion was transferred to a round-bottom flask. Next, the modified magnet with GMA was placed into this mixture and introduced into a reflux system under constant stirring at 100 °C for 48 h. After that, the resulting hybrid material (MOF@monolith) was washed with MeOH and stored in this solvent before SBSE procedure.

### Sample collection

Approximately 250 mL of water samples from different sources as tap water (Hradec Králové laboratory) and Elbe river (Hradec Králové region) were collected in dark-glass bottles and stored at 4 °C until analysis. Morning urine samples were supplied by a healthy female volunteer. Fasting urines were collected and filtered immediately before their storage at 4 °C without any further treatment.

### SBSE protocol

Before each extraction, the SBSE extraction units were firstly conditioned with 3 mL of water for 5 min. Next, the MOF@monolith stir bar was placed in 15 mL of sample and stirred for 30 min at 500 rpm. After the loading step, the stir bar was washed in 3 mL of water for 5 min. Next, the elution of retained analytes was carried out using 1.5 mL of MeOH during 30 min at 500 rpm. Subsequently, 20 µL of the eluent were directly injected into the HPLC system after filtration through 0.45-µm PTFE filter. After each protocol, the devices were cleaned twice with MeOH (5 mL × 2 for 5 min) and stored in this solvent until the next extraction.

### Chromatographic conditions

HPLC analysis was carried out using a Shimadzu Prominence LC system equipped with a fluorescence detector. All separation procedures were done using a Kinetex XB-C18 analytical column (150 mm × 4.6 mm i.d., 2.6-µm particle size). ACN (A) and water (B) were used as a mobile phase in gradient program: 0–3 min, 75–50% B; 3–7 min, 50% B; 7–7.5 min, 50–100% B; 7.5–8 min, 100% B; 8–8.5 min, 100–75% B; and 8.5–10 min, 75% B. The injection volume was 20 µL and the flow rate of the mobile phase was kept at 0.8 mL min^−1^. Column temperature was set up at 25 °C. All estrogens were monitored by fluorescence detection fixed at an excitation wavelength of 280 nm and an emission wavelength of 310 nm.

## Results and discussion

### Preliminary studies

The application of MOF materials in SBSE format has been scarcely explored [35]. In this context, the use of polymeric monoliths as substrates to immobilize MOFs is a promising approach. For this purpose, the selection of a monolith with enough mechanical and chemical stability is a key aspect to perform as SBSE device. Additionally, the host monolithic material should provide reactive groups to attach the MOF. In this sense, a poly (GMA-co-EDMA) monolith was chosen since this polymer showed the abovementioned requirements (epoxide groups, good permeability, and high stability).

On the other hand, the selection of a proper MOF is relevant since it will strongly affect the interaction with the target analytes, and, consequently, the performance of SBSE device. In this sense, several water-stable amino-modified MOFs based on MIL-101 type were selected taking into account the following characteristics: (i) the presence of amino groups in their structure allows that these materials can be attached covalently to the epoxide moieties onto the GMA monomers through nucleophilic substitution reactions [31]; (ii) the adequate MIL-101 typology in terms of pore-cage structure to host the target compounds (Fig. S1); (iii) several interaction forces between these materials and analytes such as hydrophobic effects and π-π interaction and hydrogen bonding, among others.

Next, a preliminary examination of the amino-modified MIL-101 type with different metal ions was conducted. For this purpose, three different MOFs as well as a bare polymer monolith (for comparison studies) were evaluated under identical conditions. As shown in Fig. S2, bare monolith retained a small amount of target analytes, whereas for MOFs investigated, the amino-MIL-101(Al) gave the best retention values. Bearing in mind all these considerations, the MIL-101(Al)@monolith was properly characterized prior to its evaluation as sorbent for SBSE.

### Preparation and characterization of MOF@monolith-coated stir bar

Prior to the incorporation of MIL-101(Al) to the polymer monolith, a modification of the magnet is required in order to assure a successful covalent bonding between this support and the monolith bed. This procedure based on a chemical etching of PTFE magnet by sodium naphthalenide (Fluoroetch®) followed by a vinylization process with GMA has been previously reported (see details in the “Experimental” section) [32, 33]. After PTFE magnet modification, a poly(GMA-co-EDMA) monolith, whose composition is given in the “Experimental” section, was selected as host substrate. Then, the introduction of NH_2_-MIL-101(Al) structures into the GMA-based monolith was carried on the basis of the epoxide opening reaction of the monolith and the amino groups presented in the MOF. To perform this functionalization, several organic solvents such as THF, EtOH, and DMF among others were tested as dispersing media, being DMF able to produce the most stable dispersions of these materials. Thus, the MOF@monolith in the magnet was prepared using the experimental conditions described above. These conditions provided a good compromise between the number of MOF units attached onto the monolith, extraction performance, and mechanical stability of the stirring units.

Fig. 1A–C show images of experimental design, bare monolith, and MOF@monolith immobilized onto PTFE magnet, and an optical microscope image (top view) of this extraction device (Fig. 1D). As it can be seen, the core magnet (black) is coated by a thick layer of polymer monolith (white) followed by a second thin film (yellow) corresponding to the MOF crystals. Since the monolith layer is quite larger than the resulting MOF sheet, the characterization part of this material constitutes a challenging task. Proof of this, XRD measurements of hybrid monolith were done directly on SBSE device (data not shown); however, the characteristic diffraction peaks of this MOF were not detected, even operating in Grazing incidence diffraction mode. Alternatively, a careful removal of composite coating was done and subjected to XRD analysis. In this case, small peaks of MOFs were found (Fig. S3), which is consistent with the relatively low content of MOF compared to the large contribution of the amorphous monolith in the final material (Fig. 1D)

**Fig. 2:**
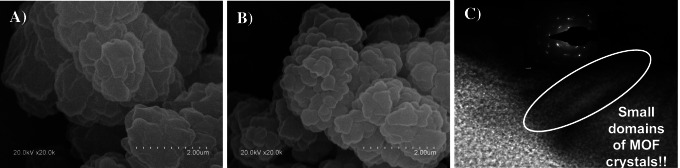
SEM micrographs of bare monolith (**A**) and MOF@monolith (**B**). HRTEM image of this hybrid material (**C**)

To corroborate what could be seen by eye, several characterization techniques were conducted to ascertain the MOF attachment onto monolith surface. SEM images (Fig. 2A–B) illustrated the resemblance in the pore shape and structure of neat polymer and MOF@monolith due to their similar morphological networks [34, 36, 37]. In this sense, HRTEM measurements were performed, where small nano-domains of MOF crystals could be observed (see Fig. 2C). Additionally, the attachment of MOF on the pore surface of polymer monolith was also demonstrated by EDX analysis. As shown in Fig. S4, the aluminum content increased from 0 (bare monolith) to 0.6% after functionalization process, which confirmed the presence of MIL-101(Al) onto the surface of magnet. Furthermore, elemental analysis of MOF@monolith was also done to evaluate the bulk MOF content. A nitrogen content of 0.82 ± 0.09 wt% attributed to amino-functionalized MOF was found. Taking into account that the nitrogen amount in the pure MOF is 8.6 ± 0.1 wt%, the percentage of MOF incorporated into the final composite can be estimated as *approx.* 9.5%. Further evidence of correct functionalization of the bare monolith with amino-modified MOF was corroborated by FT-IR. The corresponding text and figure (Fig. S5) are given in ESM.

### Optimization of SBSE extraction conditions

Once studied the characterization of hybrid material, several parameters that influence the extraction efficiency of SBSE protocol were optimized. These parameters were sample pH, salt addition, extraction time, stirring rate, desorption solvent, and time. Along the optimization study, an aqueous solution (2.5 mL) containing 500 µg L^−1^ of each EDC was used as a test mixture. All experiments were performed in triplicate. The respective text and figures (Fig. S6-7) are given in the ESM. The following experimental conditions were found to give the best results: (A) no pH adjustment; (B) no salt addition; (C) extraction time, 30 min; (D) stirring speed, 500 rpm.

Additionally, the desorption step was investigated in terms of type and volume of solvent and time. The results (Fig. S6) and detailed description are given in ESM. According to the results, 1.5 mL of MeOH and a stirring time of 30 min were chosen as the proper desorption conditions.

Once established the optimum SBSE protocol, several analytical features of hybrid material as sorbent were evaluated. In order to evaluate the breakthrough volume, different volumes (between 2.5 and 25 mL) of standard EDC solution were loaded with the stir bar material. As shown in Fig. S8, high recoveries (above 80%) up to 15 mL were obtained for all tested analytes. Large volumes (25 mL) led to a decrease in the recovery values. Therefore, 15 mL was adopted as the volume for the analysis of real water samples.

The reusability of the sorbent (see Fig. S9) was investigated by reusing the material to extract analytes using aqueous standard solutions of EDCs at 500 µg L^−1^. For this purpose, the SBSE device was repeatedly used by employing a regeneration protocol (with MeOH and water) as described above (see the “Experimental” section). The results showed that extraction device can be reused at least 7 times with extraction yields higher than 80%. Despite this acceptable reusability, further studies will be developed focusing on the increase of thickness of MOF coating in order to enhance the SBSE device reusability.

Also, a storage stability study was done with a SBSE device after 4 months of storage immersed in MeOH at room temperature. The results showed that no significant change of working capacity and extraction efficiency (> 90%) was observed over this period using aqueous standard solutions of EDCs at 25 µg L^−1^.

### Method validation

The analytical performance of the method was established using the optimum conditions found in SBSE in combination with HPLC/FLD (Table 1). Thus, the linearity and limits of detection (LOD) and quantitation (LOQ) were measured under the optimized SBSE-HPLC/FLD conditions. A good linearity (with correlation coefficient (*r*) > 0.999) was obtained in the concentration range of 2–250 µg L^−1^ for E2, EE2, and E3. For E1, the instrumental linearity (with *r* > 0.999) was in the range 50–750 µg L^−1^, due to its lower fluorescence. Using the optimized SBSE protocol, the LODs were established at signal-to-noise ratio (S/N) of 3, giving values comprised between 0.015 and 0.58 µg L^−1^, whereas the LOQs (S/N = 10) ranged between 0.05 and 1.9 µg L^−1^. The preparation reproducibility of MOF@monolith-coated stir bars was also investigated with aqueous solution samples containing 200 µg L^−1^ of each EDC. As shown in Table 1, the relative standard deviations (RSDs) of preparation reproducibility ranged from 2.2 to 6.1% (*n* = 3) in one batch and 5.1–7.1% (*n* = 3) among different batches. The preconcentration factor, calculated as the ratio comparing the peak area of the estrogens without and with SBSE treatment, ranged between 7.2 and 9.3.

**Table 1:** Analytical figures MOF@monolith magnet as SBSE sorbent in the analysis of EDCs

Estrogen	Linear range (µg L^−1^)	LOD^1^ (µg L^−1^)	Calibration plot	Enrichment factor	Precision (RSD, %)	
					Inter-day^**2**^	Inter-batch^3^
E1	50–750	0.6	*Y* = (362 ± 3) *x* − (7729 ± 1150)	9.1	3.5	5.5
E2	2–250	0.015	*Y* = (13,044 ± 131) *x* − (14,060 ± 14,793)	9.0	2.2	6.7
EE2	2–250	0.06	*Y* = (9762 ± 82) *x* − (15,263 ± 9228)	9.3	6.1	7.1
E3	2–250	0.02	*Y* = (9948 ± 93) *x* − (16,424 ± 10,448)	7.2	4.6	5.1

^1^Values obtained applying the optimized protocol

^2^Inter-day values (*n* = 3) using a single stir bar

^3^Inter-batch values (*n* = 3) using different stir bars

For RSD values, a standard concentration at 200 µg L^−1^ of each EDC was used (excitation and emission wavelengths of 280 and 310 nm, respectively)

### Analysis of EDCs in water and urine samples

**Fig. 3:**
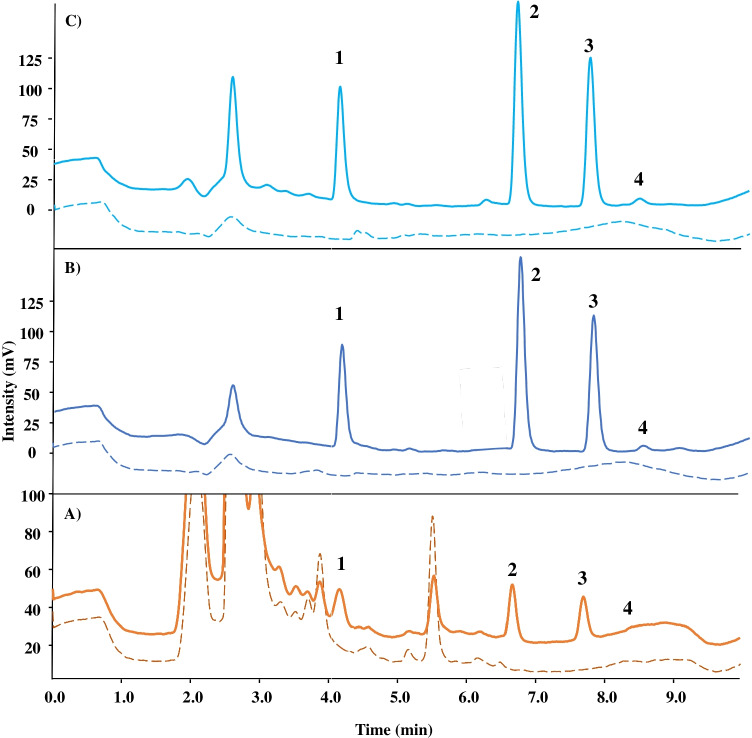
HPLC-FLD chromatograms of EDCs in (**A**) human urine, (**B**) tap water, and (**C**) Elba river water. The dashed line and continuous lines represent blank and spiked sample at 5 µg L^−1^ (urine) or 25 µg L^−1^ (water samples) after SBSE protocol, respectively. HPLC conditions: analytical column Kinetex XB-C18 (150 × 4.6 mm, 2.6 µm particle size); mobile phase ACN:water in gradient elution described in the “Experimental” section (ESM); flow rate, 0.8 mL min^−1^; injection volume, 20 µL. Peak identification: (1) E3, (2) E2, (3) EE2, (4) E1

The proposed analytical method was applied to the extraction and determination of EDCs in real samples. In water samples, none of the EDCs were found. However, estriol was detected in one of the analyzed volunteer urine samples after oral administration of hormonal contraception (see Fig. S10 and Table S2). In order to establish the accuracy of the method, both samples were spiked with the four EDCs at two concentration levels (5 and 25 µg L^−1^). Figure 3 shows the chromatograms of blank and spiked samples using the developed SBSE-HPLC/FLD method. The analytical results and the recovery for the spiked samples are listed in Table 2. The recoveries for EDCs in spiked water samples were in the range of 73–94%, whereas the spiked recoveries for urine samples were comprised between 72 and 90%.

**Table 2:** Recovery study of EDCs in spiked environmental water and human urine samples analyzed following the optimized SBSE protocol. Recovery (%) ± SD (*n* = 3)^a^

Analyte	Sample	Spiked level (µg L−1)	Recovery (%) ± SD^a^
E1	Tap water	5	–
		25	91 ± 1
	River water	5	–
		25	92 ± 3
	Urine	5	–
		25	90 ± 4
E2	Tap water	5	79 ± 2
		25	94 ± 2
	River water	5	88 ± 7
		25	91 ± 3
	Urine	5	86 ± 1
		25	85 ± 6
EE2	Tap water	5	87 ± 4
		25	91 ± 1
	River water	5	87 ± 6
		25	94 ± 1
	Urine	5	89 ± 3
		25	90 ± 3
E3	Tap water	5	83 ± 4
		25	73 ± 3
	River water	5	79 ± 4
		25	76 ± 4
	Urine	5	72 ± 2
		25	78 ± 3

### Comparison with commercial PDMS stir bars and other extraction protocols

The proposed SBSE-HPLC/FLD method was firstly compared with commercial PDMS stir bars under the best SBSE conditions. As shown in Fig. S11, the extraction performance of our SBSE device was much higher than that found using the PDMS-coated stir bar, which can be attributed to the features (low selectivity and long extraction times to reach the equilibrium conditions) of this commercial sorbent. This result was consistent with previous studies [25, 38]. Besides, a comparison with other sample preparation approaches [5, 7, 38–41] for the extraction of EDCs in environmental water and biological samples was accomplished (Table 3). In general, the developed method showed similar sample volumes and pretreatment times than other works reported in the literature [38, 40, 41]. With regard to the recovery values in particular, except from the some works [5, 39], these were similar to those obtained in most reported studies [7, 38, 40, 41]. The present method showed better precision (RSD values below 7%) than other SBSE studies [5, 38, 39] or vortex-assisted membrane extraction [39] and similar to other microextraction methods [7, 40]. Concerning LODs, the MOF@monolith method provided similar values to other methods [41] even when high sensitive and sophisticated technique (like UPLC-MS/MS) was used [40]. In any case, the developed method offered lower LODs than SBSE [38, 39] and magnetic SPE [5]. Besides, our SBSE method avoids the centrifugation steps (commonly used in dispersive SPE or UA) [5, 7, 40], thus reducing losses of material or analytes during operation. Another strength of our SBSE support is that the PTFE-based magnet showed a large mechanical resistance compared to the fragility of the typical PDMS substrate (thin glass jacket with an incorporated magnet core) vulnerable to breaking during the handling.

**Table 3:** Comparison between the developed SBSE-HPLC-FLD procedure and similar methods reported in the literature

Analytes	Method	Material	Sample matrix	Sample Volume (mL)	Pretreatment time (min)^a^	Recoveries (%)	RSD (%)	LODs (µg L−1)	Ref
EE2	MSPE-HPLC–UV	AC/Fe_3_O_4_	water	0.75	4	57	≤ 15	800	[5]
E1, E2, E3	d-MSPE	Fe_3_O_4_@ZnAl-LDH/MOF	milk	1	14.5	72–90	≤ 8	0.003–0.005	[7]
E1, E2, EE2	SBSE-HPLC–UV	MOF@PDMS	water	10	55	88–124	≤ 16	0.3–0.4	[38]
E1, E2, EE2	SBSE-HPLC–UV	PDMS	water, urine	30	120	11–25	≤ 17	0.3–1.0	[39]
E1, E2, EE2, E3	d-MSPE-UPLC-MS/MS	MOF	water, urine	8	70	80–107	≤ 10	0.03–1.0	[40]
E1, E2, EE2, E3	VA-ME-HPLC-FLD	MOF-MMMs	urine	20	45	81–103	≤ 11	0.005–1	[41]
E1, E2, EE2, E3	SBSE-HPLC-FLD	MOF@monolith	water, urine	15	60	72–94	≤ 7	0.015–0.6	This work

*MSPE* magnetic solid-phase extraction, *d-MSPE* dispersive-MSPE, *SBSE* stir bar sorptive extraction, *VA-ME* vortex-assisted membrane extraction, *FLD* fluorescence detection, *AC* activated carbon, *LDH* layered double hydroxide, *PDMS* polydimethylsiloxane, *MOF* metal–organic framework, *MISPE* molecularly imprinted solid-phase extraction, *MMMs* mixed-matrix membranes

^a^Evaporation time for the injection into the HPLC system is not taken into account

## Conclusions

In this work, the first example of polymer monolith modified with MOF onto commercial PTFE magnets for SBSE purposes is presented. The resulting home-made stir bars with the MOF@monolith were evaluated to extract EDCs. The presence of the MOF in the hybrid material has demonstrated an enhanced retention of these compounds owing to hydrophobic, π-π interaction, and hydrogen bonding interactions, showing better extraction performance than commercial PDMS stir bars. The developed stir bar coating demonstrated several advantages such as cost-effective fabrication, excellent preparation reproducibility, and acceptable reusability. Besides, the combination of this coating with HPLC/FLD was satisfactorily applied to the extraction and determination of EDCs in environmental water and human urine samples. This work demonstrates for the first time that the combination of MOFs, monoliths, and SBSE is possible in a rather simple manner and thus it can be an attractive possibility in sample preparation, and it opens new areas for advanced extraction approaches.

## Supplementary Information

Below is the link to the electronic supplementary material.

Supplementary file1 (DOCX 10041 KB)
