# Supplementary material for: Development of hybrid monoliths incorporating metal–organic frameworks for stir bar sorptive extraction coupled with liquid chromatography for determination of estrogen endocrine disruptors in water and human urine samples
Source: Mikrochim Acta. 2022 Feb 7;189(3):92. doi: 10.1007/s00604-022-05208-6 (PMC8821068; doi:10.1007/s00604-022-05208-6)
Supplement: Supplementary file 1 — Supplementary file1 (DOCX 10041 KB) [file 604_2022_5208_MOESM1_ESM.docx]

**Electronic Supporting Material on the Microchimica Acta publication entitled**

Development of hybrid monoliths incorporating metal-organic frameworks for stir bar sorptive extraction coupled with liquid chromatography for determination of estrogen endocrine disruptors in water and human urine samples

S. Zatrochová^1+^, H. Martínez-Pérez-Cejuela^2+^, M. Catalá-Icardo^3^, E.F. Simó-Alfonso^2^, I. Lhotská^1^, D. Šatínský^1^, J.M. Herrero-Martínez^2^*

*^1^Department of Analytical Chemistry, Faculty of Pharmacy in Hradec Králové, Charles University, Ak. Heyrovského 1203, Hradec Králové 500 05, Czech Republic*

*^2^Department of Analytical Chemistry, University of Valencia, Dr Moliner 50, 46100, Burjassot, Valencia, Spain*

*^3^Instituto de Investigación para la Gestión Integrada de Zonas Costeras, Campus de Gandía, Universitat Politècnica de València, C/ Paranimf 1, 46730 Grao de Gandía, Valencia, Spain*

^+^Both authors contributed equally to this manuscript.

**Table of contents**

Page S1-S3. Experimental section

Page S3-S5. Results and discussion

Page S6. EDC Structures and physicochemical properties (Table S1)

Page S7. Structural sizes of EDCs and MOF cavities (Figure S1)

Page S8. Influence of metal nature of MOF on the recoveries (Figure S2)

Page S8. p-XRD patterns from bare monolith, MOF and hybrid material (Figure S3)

Page S9. EDX analysis from MOF@monolith device (Figure S4)

Page S9. FT-IR spectra from bare monolith, MOF and hybrid material (Figure S5)

Page S10. Optimization protocol (Figure S6)

Page S11. Influence of ionic strength on the retention (Figure S7)

Page S11. Influence of sample volume on the EDC recoveries (Figure S8)

Page S12. Reusability study of the extractant units (Figure S9)

Page S12. EDC quantities in urine samples (Table S2)

Page S13. HPLC-FLD chromatogram from urine samples (Figure S10)

Page S14. Comparison between commercial PDMS stir bars and our lab-made devices (Figure S11)

Page S15. References

# Experimental section

*Reagents and materials*

PTFE-coated stirring bars (15 mm length × 1.5 mm diameter) were obtained from VWR International Eurolab (www.vwr.com, Barcelona, Spain). Sodium naphthalene solution (FluoroEtch^®^) for the treatment of PTFE stir bar surface was provided by Acton Technologies (http://www.actontech.com, County Limerick, Ireland). Commercial PDMS coated stir bars (Twister; Gerstel, Mullheim a/d Ruhr; Germany) with 20 mm in length and 0.5 mm film thickness were used for SBSE comparison purposes. Glycidyl methacrylate (GMA) and ethylene glycol dimethacrylate (EDMA) were from Sigma-Aldrich (www.sigmaaldrich.com, Steinheim, Germany). Lauroyl peroxide (LPO), cyclohexanol and 1-dodecanol were from Alfa-Aesar (www.alfa.com, Karlsrube, Germany). Fluorinated ethylene-propylene (FEP) tubing (3.60 × 6.00 mm) was provided by Bohlender GmbH (https://www.bohlender.de, Grünsfeld, Germany).

Aluminum and iron trichloride hexahydrate, chromium trinitrate nonahydrate, 2-aminoterephtalic acid (99%) were purchased from Sigma-Aldrich. N,N-dimethylformamide (DMF) from VWR Chemicals (Fontenay Sous Bois, France). β-estradiol (E2) (≥98%), 17β-ethinylestradiol (EE2) (≥98%) were from Sigma-Aldrich. Estrone (E1) and estriol (E3) (≥95%) were acquired from Cayman (https://www.caymanchem.com, Michigan, USA). The physicochemical properties of EDCs are shown in Table S1. Sodium phosphate monobasic monohydrate (≥98%) were obtained from Thermo Fisher (https://www.thermofisher.com, Kandel, Germany). Tetrahydrofuran (≥99.0%) and ethanol were purchased from Sigma-Aldrich. HPLC-grade methanol (MeOH), acetonitrile (ACN), dimethylformamide, ethyl acetate (≥99.5%), and acetone were from VWR.

Stock solutions of the EDC standards were prepared in methanol at concentration of 1 mg mL^-1^ and stored at 4°C. Mixed standard solutions were prepared daily prior to use. All other reagents were of analytical grade unless otherwise stated. Deionized water was prepared in the Crystal B30 EDI Adrona deionizer (www.adrona.lv, Riga, Latvia).

*Instrumentation*

OptikamB5 Digital camera coupled to magnifying glass (https://www.optikamicroscopes.com, Optika, Ponteranica, Italy) was used to observe the MOF layer formation onto the magnet.

Scanning electron microscopy (SEM) micrographs were acquired with a Hitachi S-4800 electron microscope (http://www.hitachi.com, Ibaraki, Japan) provided with a retrodispersive electron detector and an energy dispersive spectrometer (EDAX Genesis 4000). For high resolution transmission electron microscopy (HRTEM), a carbon coated nickel microgrid was used, where a dispersion of the samples in ethanol were deposited. Prior to image acquisition, an air stream was used to dry the samples. HRTEM images were carried out in a JEOL microscope (JEM 2100F, Hitachi) set at 200 kV.

Diffraction (p-XRD) patterns operating with Bragg-Brentano geometry and Grazing incidence diffraction (GID) measurements were obtained in a D8 Advance A25 diffractometer (Bruker Daltonik GmbH, Bremen, Germany) with experimental conditions of 40 mA and 40 kV in each analysis. A single diffractogram acquired at room temperature from 2θ = 2° to 2θ = 50° is the average of five repeated measurements.

Also, attenuated total reflection Fourier-transform infrared (FT-IR) spectra of powdered materials were registered with a DuraSamplIR II accessory from Smiths Detection Inc. (www.smithsdetection.com, Warrington, UK) equipped with a nine reflection diamond/ZnSe DuraDisk plates, installed on a Bruker FT-IR spectrometer (https://www.bruker.com, Bremen, Germany) model Tensor 27. Elemental analysis was carried out with an EA 1110 CHNS elemental analyzer (CE Instruments, Milan, Italy).

Chromatographic separation of hormones, determination, and method validation were carried out on a Shimadzu Prominence (https://www.shimadzu-la.com, Shimadzu Corporation, Kyoto, Japan) HPLC system equipped with RF-10A XL fluorescence detector. The system consisted of an LC-20 AD dual-pump module, a DGU-AS mobile phase degasser, SIL-20 AC autosampler, and a CTO-20AC column oven. The HPLC system was controlled by a CBM-20A communication module. Lab-Solution software (Shimadzu Corporation) was used for the data acquisition and evaluation.

# Results and discussion

*Preparation and characterization of MOF@monolith coated stir bar*

In order to further confirm the correct synthesis of the final composite, FT-IR spectra was adquired (Fig. S5) and the characteristic peaks corresponding to NH_2_-MIL-101(Al) structure from 750 to 1600 cm^-1^ can be observed in the MOF@monolith device with lower intensities. Recognizable peaks from C-N stretching absorption of aromatic amines (1335-1250 cm^-1^) and characteristic vibrational bands of the framework (O-C-O) groups around 1400 cm^-1^ and 1550 cm^-1^ are present in both MOF and hybrid material. Also, the signal centrered at 1750 cm^-1^, present in the bare monolith and the composite, can be related to the stretching vibration (C=O), which was as a result of GMA and EDMA bond. All these data reinforced the successful MOF attachment onto the surface of GMA-based monolith.

## *Optimization of SBSE extraction conditions*

Structurally, EDCs show phenolic hydroxyl groups, and their existing forms in the aqueous solution can be affected by the pH value. Therefore, the effect of this variable on extraction efficiency of these analytes was investigated within the pH range of 3.0-9.0. As shown in Fig. S6A the pH had no obvious effect on the extraction of target estrogens in the investigated range. Higher pH values than 9.0 were not tried since a decrease in extraction was expected due to the ionization of EDCs (pK_a_ values of EDCs about 10.4 [1, 2]). From the above results, it can be concluded that the extraction of analytes is favored when they were present in their molecular form, being favorable their retention by hydrophobic effects, π-π interactions and hydrogen bonding. Thus, according to the usual pH range found in surface water systems (6.5 to 8.5) and urine samples (4.5 to 8.0), no pH adjustment was done for further studies.

Another parameter that influences extraction efficiency is the salt concentration, which can affect the interactions of the analytes toward the MOF@monolith. As illustrated in Fig. S7, the retention performance was almost similar up to 2.5% (w/v), whereas at 5% (w/v) the retention of EDCs decreased. This decrease could be explained by a possible interaction of polar EDCs with the salt molecules, resulting in a decrease of the extraction efficiency [2, 3]. Taking into account these results, no salt was added to the samples.

Extraction time is a key factor affecting the extraction efficiency. The effect of this variable in the range of 15-60 min was therefore also investigated (Fig. S6B). Experimental results showed that extraction equilibrium for four estrogens was reached at 30 min, remaining the extraction efficiency almost unchanged after this time. Therefore, this value was selected for the following experiments.

Sample stirring has a major role to increase the extraction efficiency by improving the mass transfer of target analytes toward the sorbent and decrease the extraction time. The effect of stirring rate was studied in the range of 200 to 800 rpm (Fig. S6C). The obtained results revealed that a stirring rate up to 500 rpm was considered adequate to achieve satisfactory retention of analytes, and it was selected for further experiments. High stirring rate affected negatively the extraction of analytes.

The selection of an appropriate eluting solvent for the retained EDCs from the stir bar was firstly studied. For this purpose, different solvents including ACN, MeOH, acetone, and ethyl acetate were tested (Fig. S6D). The experimental data showed that the best recoveries were obtained using MeOH as desorption solvent. It can be due to that MeOH can easily enter the MOF pores interacting with the analytes and release them easily. Thus, this solvent was selected for the remaining experiments.

The desorption volume can have effect on the enrichment factor, waste generation, and organic solvent consumption (Fig. S6E). In order to study the effect of desorption volume on the performance of the method, different volumes of MeOH from 0.5 to 2.5 mL were investigated. As shown in Fig. S6E, the volume could be reduced up to 1.5 mL being this volume enough to desorb the target analytes with satisfactory extraction performance.

Also, the effect of desorption time on the extraction efficiency was studied from 15 to 45 min for the desorption of the target analytes (Fig. S6F). The results showed that the desorption process reached an equilibrium after 30 min of contact time being this time chosen for subsequent experiments.

**Table S1.** Structures and physicochemical properties of endocrine-disruptor compounds (EDCs).

| **Compound** | **Molecular formula** | **Structure** | **Log K_ow_^1^** | **pK_a_^2^** |
| --- | --- | --- | --- | --- |
| Estrone (E1) | C_18_H_22_O_2_ |  | 3.13 | 10.34 |
| 17-β-estradiol (E2) | C_18_H_24_O_2_ |  | 4.01 | 10.23 |
| 17-β-ethinylestradiol (EE2) | C_20_H_24_O_2_ |  | 3.67 | 10.25 |
| Estriol (E3) | C_18_H_24_O_3_ |  | 2.45 | 10.25 |

^1^Values of Log K_O/W_ were obtained from Hazardous Sbstances Data Bank (PubChem, 14/09/2021); ^2^values of pKas were found from Schäfer *et al*.[4]


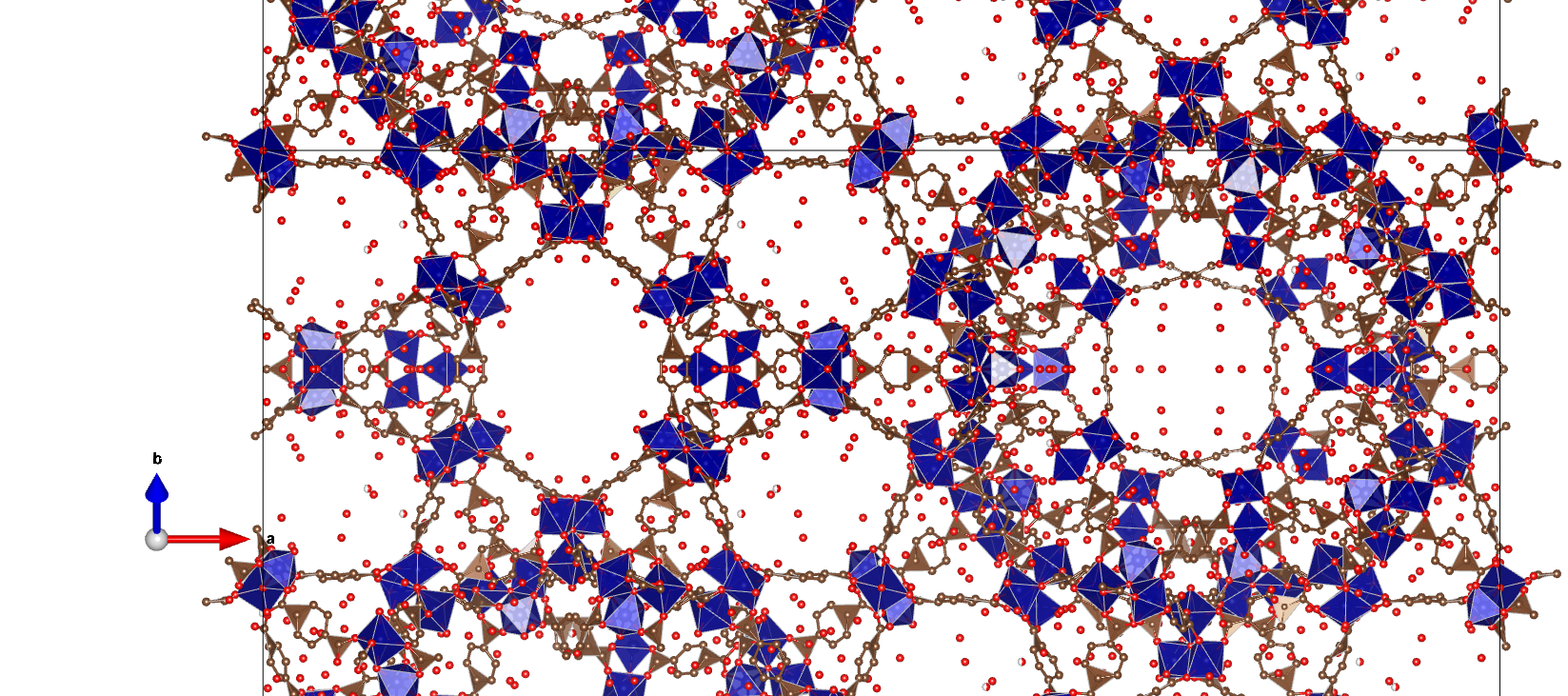


**~16 Å**

**~ 12 Å**


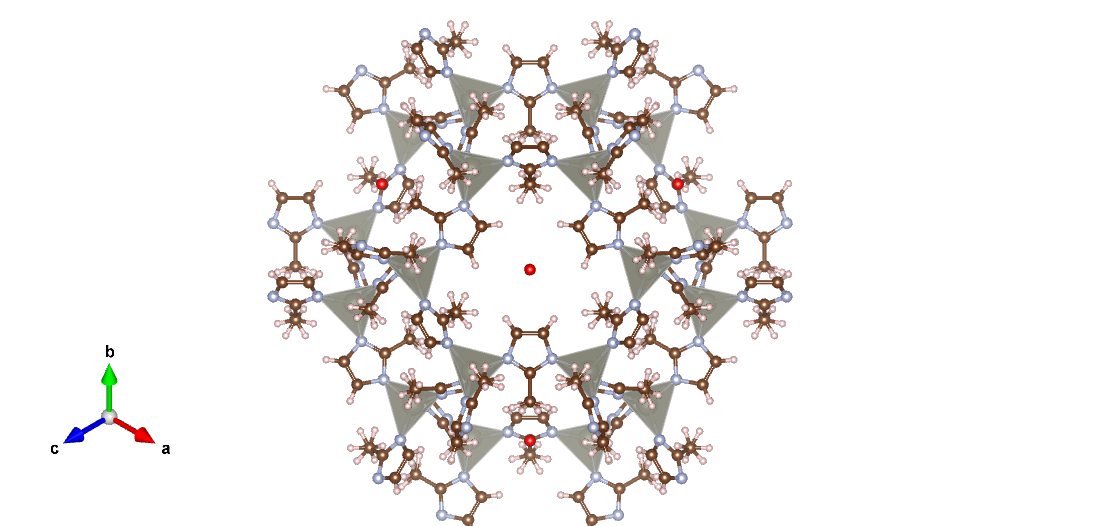


E1

10.8 Å

5.3 Å

1.5 Å

E3

11.2 Å

5.3 Å

1.5 Å

E2l

10.9 Å

5.3 Å

1.5 Å

EE2

10.9 Å

4.4 Å

5.0 Å

**Figure S1.** Molecular sizes of the EDC molecules and MIL-101(Al). Colors’ code: O atoms (red), C atoms (brown) and Al atoms (dark blue inside of tetrahedrals). The structures of analytes were obtained from Chem3D. To obtain the sizes, an energetic minimization was done using MM2 model at 300K. (Minimum RMS Gradient = 0.0100).

**Fig. S2.** Preliminary study of MOF metal nature and its influence on the recoveries. Error bar = SD (n=3). The rest of conditions are given in the Experimental section.

**Fig. S3.** Experimental p-XRD patterns of bare monolith (red), NH_2_-MIL-101(Al) (black) and MOF@monolith (grey).


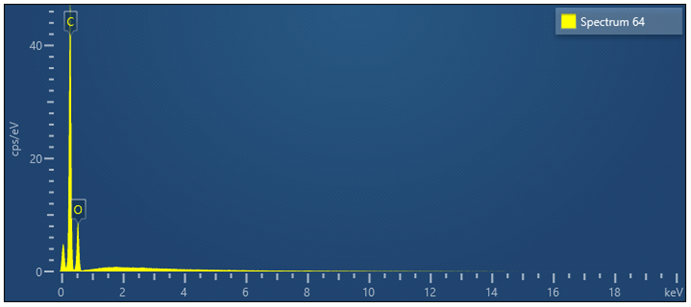


**Bare monolith**


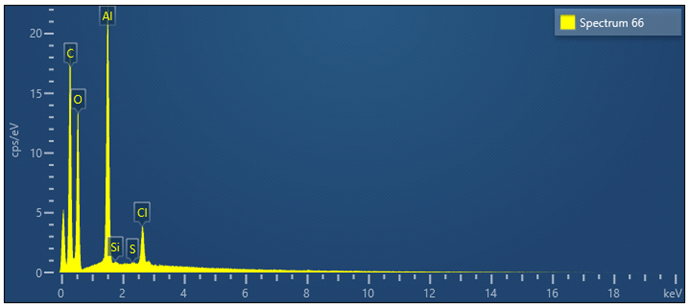


**NH_2_-MIL-101 (Al)**


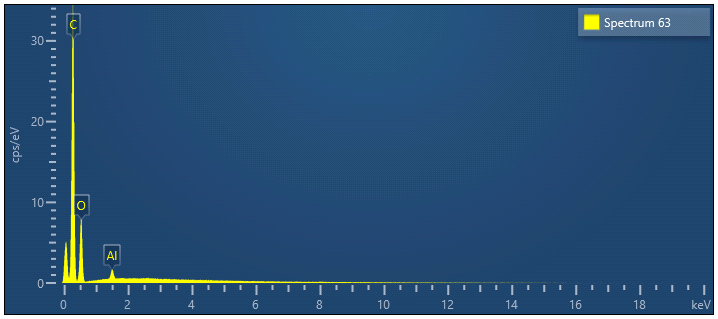


**NH_2_-MIL-101(Al)@monolith**

**Aluminum content**

**0 % (bare monolith)**

**0.6 % (2 days synthesis)**

**Fig. S4.** EDX analysis of the studied materials including the Al content in the final hybrid material.

**Fig. S5.** FT-IR spectra of individual materials and the final composite (MOF@monolith SBSE device).

**Fig. S6.** Effect of the pH (a), extraction time (b), stirring rate (c), desorption solvent vortex time (d), desorption volume (e), and desorption time (f) on the extraction recoveries for the target EDCs. Experimental conditions (except the parameter under investigation): sample volume, 2.5 mL aqueous standard solution (no salts and pH adjustment): extraction and desorption time, 30 min; stirring rate, 500 rpm; desorption eluent, 2.5 mL MeOH.

**Fig. S7.** Influence of sodium salt (ionic strength) on the retention performance of MOF@monolith material. Error bar = SD (n=3). The rest of conditions are given in the Experimental section.

**Fig. S8.** Breakthrough volume study using the hybrid material device. Error bar = SD (n=3). The rest of conditions are given in the Experimental section.

**Fig. S9.** Reusability graph of the final magnets. Error bar = SD (n=3). The rest of conditions are given in the Experimental section

**Table S2.** EDC amount found in the analysis of 4 fresh morning urines.

| **Volunteer** | **E1** | **E2** | **EE2** | **E3** |
| --- | --- | --- | --- | --- |
| **1** | <LOD | <LOQ | <LOD | <LOD |
| **2** | <LOD | <LOQ | <LOD | <LOD |
| **3** | <LOD | <LOD | <LOD | <LOD |
| **4** | <LOD | <LOD | <LOD | 7.49 µg L^-1^ |

.

**0**

**1**

**2**

**3**

**4**

**5**

**6**

**7**

**8**

**9**

**Time (min)**

**50**

**100**

**150**

**200**

**Intensity (mV)**

**1**

**2**

**3**

**4**

**Fig. S10.** HPLC-FLD chromatograms of EDCs in fresh woman urine nonspiked (black line) and spiked at 5 µg L^-1^ after SBSE protocol, respectively. HPLC conditions: analytical column Kinetex XB-C18 (150 × 4.6 mm, 2.6 µm particle size); mobile phase ACN:water in gradient elution described in Experimental section (ESM); flow rate, 0.8 mL min^-1^; injection volume, 20 µL. Peak identification: 1) E3, 2) E2, 3) EE2, 4) E1.

**2**

**3**

**4**

**1**

**Fig. S11.** Comparison of HPLC-FLD chromatograms of EDCs with standard at 25 µg L^-1^ under the optimum conditions (see Experimental section). Black and red lines depict the recovered areas from SBSE protocol using the developed MOF@monolith and commercial PDMS device, respectively. HPLC conditions: analytical column Kinetex XB-C18 (150 × 4.6 mm, 2.6 µm particle size); mobile phase ACN:water in gradient elution described in Experimental section (ESM); flow rate, 0.8 mL min^-1^; injection volume, 20 µL. Peak identification: 1) E3, 2) E2, 3) EE2, 4) E1.

**References**

1. Hurwitz AR, Liu ST (1977) Determination of aqueous solubility and pKa values of estrogens. J Pharm Sci 66:624-627. <https://doi.org/10.1002/jps.2600660504>
2. Hu C, He M, Chen B, Zhong C, Hu B (2013) Polydimethylsiloxane/metal-organic frameworks coated stir bar sorptive extraction coupled to high performance liquid chromatography-ultraviolet detector for the determination of estrogens in environmental water samples. J Chromatogr A 1310:21-30. <https://doi.org/10.1016/j.chroma.2013.08.047>
3. Lord H, Pawliszyn J (2000) Microextraction of drugs. J Chromatogr A 902:17-63. <https://doi.org/10.1016/S0021-9673(00)00836-0>
4. Schäfer IA, Akanyeti I, Semião JA (2011) Micropollutant sorption to membrane polymers: a review of mechanisms for estrogens. Adv Colloid Interface Sci, 164: 100-117.
